# Differential expression of complement Properdin and Factor H in the placentae and umbilical cords of mothers with Preeclampsia, Gestational Diabetes Mellitus and Recurrent Pregnancy Loss

**DOI:** 10.3389/fimmu.2025.1731251

**Published:** 2026-02-03

**Authors:** Tamali Roy, Ananya Das, Nabanita Chatterjee, Shyama Prasad Saha, Rajib Prasad, Taruna Madan, Hadida Yasmin, Uday Kishore

**Affiliations:** 1 Department of Zoology, Cooch Behar Panchanan Barma University, Cooch Behar, West Bengal, India; 2 Department of Receptor Biology and Tumor Metastasis, Chittaranjan National Cancer Institute, Kolkata, West Bengal, India; 3 Department of Obstetrics and Gynaecology, Maharaja Jitendra Narayan Medial College and Hospital, Cooch Behar, West Bengal, India; 4 Hospital Administration & Medical Services Department, Maharaja Jitendra Narayan Medical College & Hospital, Cooch Behar, West Benga, India; 5 Clinical Studies and Trials Unit (CSTU), Division of Development Research, Indian Council of Medical Research, (ICMR), New Delhi, India; 6 Department of Veterinary Medicine (CAVM), United Arab Emirates University, Al Ain, United Arab Emirates

**Keywords:** complement, properdin, factor H, pregnancy, preeclampsia, gestational diabetes mellitus, recurrent pregnancy loss, placenta

## Abstract

**Background:**

Properdin and factor H (FH), the two regulatory proteins of the alternative complement pathway, oppose each other to maintain the complement system’s activation. While properdin upregulates, FH downregulates the complement alternative pathway. The current study evaluated the expression of properdin and FH transcripts and proteins in the placental tissues and umbilical cords (UC) of preeclampsia (PE), gestational diabetes mellitus (GDM), and recurrent pregnancy loss (RPL) compared to normal healthy pregnancy (N).

**Methods:**

The tissue histology of PE, GDM and RPL were observed using haematoxylin-eosin and Masson’s trichrome staining. To understand the expression and distribution of properdin and FH, RT-qPCR, western blot, and immunohistochemistry were carried out. The expressions of two additional complement components, C3 and C5, were also detected by western blot.

**Results:**

The placentae from PE and GDM showed substantial collagen and fibrinoid deposition, thicker foetal blood capillaries, and a considerable number of syncytial knots. There was a significant rise in the level of properdin and significant decline in the level of FH at both mRNA and protein levels in the placentae and umbilical cord of PE compared to N; in GDM placentae, both properdin and FH were significantly elevated compared to N. In the case of RPL placentae, similar to PE, properdin expression was high while FH expression level was low. In both PE and RPL placentae, C3 and C5 levels were high, suggesting possibility of overactivation of complement proteins in the placenta.

**Discussion:**

The observed elevated properdin level can contribute to the heightened inflammatory response in PE, GDM and RPL placentae. Low FH and high C3 and C5 in the placenta possibly suggests dysregulated complement activation in PE and RPL.

## Introduction

Human placenta is an extraembryonic selective barrier between the foetus and the mother, acting as a conduit system supporting the growing foetus. Placenta, in conjunction with the umbilical cord, serves as a vital interface for the exchange of gases, nutrients, and waste products between maternal and foetal circulation, thereby supporting optimal foetal development (1–3). Foetal villi are bathed in the maternal blood, and thus, stress-related factors such as maternal circulating inflammatory cytokines (4), oxidative stress markers (5, 6), Damage-associated molecular patterns (DAMPs) (6), and endocrine/metabolic mediators (7) released in the maternal circulation during pathogenic assault can have detrimental effects leading to adverse pregnancy outcomes. Despite these adverse conditions, the establishment of immune privilege at the maternal-foetal interface, along with coordinated vascular remodelling and cellular adaptations, ensures the successful continuation of pregnancy (8–11).

Complement is one of the important innate immune systems, which plays a vital role in pregnancy and parturition (12, 13). There are more than 50 membrane-bound and soluble components of complement system which are involved in protection against invading pathogens and can generate membrane attack complex (MAC) for lysis, via three independent pathways– classical, lectin and alternative (14). Some of the complement components can also offer defence against pathogens without participating the complement pathway (15, 16). Despite being mostly derived from the liver (17), complement components are also synthesised at the extra-hepatic locations in different tissues and organs (18). In the cervico-vaginal mucosa, mannan-binding lectin (MBL) and C3 can bind to bacteria colonizing on clue cells (19, 20). As a physiological response, complement activity increases in pregnant women, and thus, there is a rise in the circulating complement proteins in the plasma. A prospective study carried out by He et al. showed that serum C3 and C4 levels increase gradually as the pregnancy progresses while C1q, C5a and sC5b‐9 levels remain similar to non‐pregnant condition in the plasma (21). The C-reactive protein (CRP), C4d, C3a and C9 levels are high whereas C1-inhibitor is low in the circulation in pregnant subjects compared to non-pregnant women (22). In the first trimester placenta, C1q, C3 and C4 are localized mostly in the fibrin and fibrinoid areas (23); C1q, C4, C3, C3d, C5, C6 and C9 are present in both pre-term and term placentae in varied locations (24), suggesting the relevance of complement proteins in a healthy pregnancy. Several mechanistic studies involving placental cell lines and gene knock-out mice, and observational studies in human samples have suggested that complement proteins play key roles in immune protection, inflammation regulation, immune tolerance, and host-pathogen interactions; in addition, they also support pre-implantation (25–27), implantation (12, 28), vascular remodelling (29, 30) placental development (28), trophoblast invasion (29, 31–33) and parturition (19). Thus, dysregulated complement activation, either inadequate or overstimulated, can lead to pregnancy complications.

Nearly all the biological consequences of complement subcomponents are dependent on the resultant cleavage products, and their levels have been found to be altered in adverse pregnancies (34). Increased deposition of C1q, C3d and C9 in chorionic villi (33), high C4d (35), and C3 and C4BP localization in syncytial bodies (32) have been observed in preeclampsia (PE) placentae. Increased levels of complement components such as factor B (36), C3b (37), C5a (38, 39), terminal complement complex (sC5b-9) (40, 41), MBL (42), C4d (35), H-ficolin, L-ficolin (43), C5a (44), aberrant C3a-C5a serum level (45) and decreased levels of C1q (32) were also observed in PE. Similarly, elevated levels of C4d placental deposition in unexplained recurrent miscarriage (46), Bb activation in early pregnancy and spontaneous preterm birth (47), and increased serum mannan-binding lectin-associated serine proteases (MASP1 and MASP2) (48) were associated with gestational diabetes mellitus (GDM). Thus, a range of studies appear to indicate dysregulated/altered complement activation in pregnancy-related complications; however, research gap exists in understanding factors and circumstances that can influence complement activation.

Local control by surface-bound and soluble complement regulators is critical to prevent complement-mediated tissue damage in normal pregnancy. An optimal degree of continuous low- grade complement activation is thus maintained and regulated at the level of initiation, amplification, and generation of effectors such as opsonin, MAC, and pro-inflammatory anaphylatoxins by complement regulators (49–54). Among the complement regulators, properdin and factor H (FH) are two vital soluble complement proteins that are secreted in the maternal circulation and in the placenta, tightly controlling the complement activation (54–56). Properdin, 53 kDa glycoprotein, which is dispersed in plasma at a concentration of about 25µg/ml (55) in a 26:54:20 ratio (55, 57), is the only upregulator of complement alternative pathway (AP) and can induce inflammation (58, 59), resulting in tissue damage (60, 61), immune cell infiltration (62) and pro-inflammatory cytokine release (63, 64). Properdin binds to and stabilizes the alternative pathway C3 convertase, C3bBb, extending its half--life by 5- to 10-fold, and enhances deposition of C3b, and thus, increasing the alternative pathway amplification loop (55, 65). Complement Factor H (FH), a 155 kDa soluble protein present in plasma in the range of 116 to 562 μg/ml, is a key negative regulator of the complement alternative pathway that promotes the proteolytic breakdown of C3b, downregulating the alternative pathway (66–69). FH influences the C3bBb convertase in two ways: it competes with factor B for binding to C3b, thus preventing formation of the C3bBb convertase, and it also accelerates the decay of this convertase once already formed. In addition, FH regulates the C3b-containing C5 convertases and acts as a cofactor for factor I and inactivates C3b (68–72). FH also inhibits complement from being amplified on target cells and on the host tissues’ extracellular matrix, preventing complement-mediated tissue damage and minimizing pro-inflammatory response that leads to a range of diseases (73–78).

Both properdin and FH perform key functions as complement regulators and as modulator of several cellular immune functions (55). Although a complex protective microenvironment exists at the maternal-foetal interface, maternal health can often influence the homeostasis of this communicating barrier compromising the foetal health under pathological conditions. The balance between properdin and FH is therefore crucial at the maternal–foetal interface, where complement must defend against pathogens without damaging foetal tissues. Thus, the current study examines the expression, distribution and localization of these two opposing complement alternative pathway regulators in adverse pregnancy cases (PE, GDM and RPL) in the placentae as well as in the umbilical cords that serves as the primary conduit between the mother and the foetus. The properdin and FH expressions were examined at gene as well as protein levels via RT-qPCR, western blot (WB) and immunohistochemistry (IHC). In addition, to understand the possible connection with complement dysregulation, local levels of C3 and C5 were also assessed. This study provides a novel insight into the potential role of properdin and FH in adverse pregnancy, thus, offering new avenues for the development of biomarkers for early detection risk stratification and/or therapeutic targeting in PE, GDM and RPL.

## Materials and methods

### Study design and sample collection

The current study was undertaken to evaluate the importance of properdin and factor H in PE, GDM and RPL for diagnostic and therapeutic intervention. Pregnant women between the age of 20 and 30 years admitted to Maharaja Jitendra Narayan Medical College and Hospital (MJNMCH), Cooch Behar, West Bengal, India, between the year 2020 and 2024 were recruited for the study. Four study groups were considered in this study: Group 1/Case group I/Preeclampsia (PE) (n=6), Group 2/Case group II/Gestational diabetes mellitus (GDM) (n=4), Group 3/Case group III/Recurrent pregnancy loss (RPL) (n=4) and Group 4/Control group/Normal healthy pregnancy (N) (n=6).

PE was diagnosed in accordance with the American College of Obstetricians and Gynaecologists (ACOG) guidelines (79). The inclusion criteria for Group 1/PE are new onset of hypertension where systolic ≥ 140mmHg and diastolic ≥ 90mmHg at two occasions at least 4 h apart after 20^th^ week of gestation, or a systolic ≥ 160mmHg and/or a diastolic ≥ 110mmHg within short interval, or proteinuria ≥ 300 mg in a 24 h urine collection, or a protein/creatinine ratio ≥ 0.3 or a dipstick reading of ≥ 1+ after 20^th^ week of gestation and in the absence of proteinuria new onset of any among, thrombocytopenia (Platelet count <1,00,000/µL), renal insufficiency, pulmonary edema, impaired liver function, or cerebral/visual symptoms. GDM is the onset or first recognition of glucose intolerance during pregnancy. Group 2/GDM mothers were considered for the study following OGTT (75 g oral glucose tolerance test) with fasting PG≥5.1 mmol/l, 1-h PG≥10.0 mmol/l, and 2-h PG of 8.5–11.0 mmol/l. Those who were diagnosed with diabetes mellitus or prediabetes (impaired fasting glucose or impaired glucose tolerance) before pregnancy or unable to complete OGTT (oral glucose tolerance test) by 32-week gestation were excluded from the study. Inclusion and exclusion criteria of GDM were followed as per the HAPO (Hyperglycaemia and adverse pregnancy outcomes) Study Cooperative Research Group and International Association of Diabetes and Pregnancy (IADP) Study Groups Consensus Panel (80, 81). For group 3/RPL, the criteria considered were continuous two or more episodes of the natural loss of pregnancy prior to the 20th gestational week of pregnancy and no previous successful pregnancy with the same partner. Patients with previous venous or arterial thrombosis or a family history of thromboembolism were also excluded. For RPL, guidelines were followed as mentioned in ACOG, 2018 and by the practice Committee of the American Society for Reproductive Medicine (82–84). For group 4/Control group, normal pregnant women (N) were matched controls (with the cases) with regards to age and gravida (number of pregnancy). Healthy pregnant mothers with no hypertension, no proteinuria, non-diabetes, and with no chronic disease or therapy, were included in this group. Women with any form of ailment or disease mentioned for the group 1/2/3 or with hypertension observed after 3 months of delivery were excluded. The present study excluded all subjects with the following: a history of antibiotic use longer than 7 days, with thyroid disease, cancer, mental illness, premature birth history, intrahepatic cholestasis during pregnancy, hypertensive disorder complicating pregnancy, smokers, previously known systemic disease, multifetal gestation, conception using gonadotropin ovulation induction or by *in vitro* fertilization. This investigation also excluded pregnant women having either HELLP syndrome (haemolysis, high liver enzymes, and low platelet count), or eclampsia. The clinical data on the human subjects are summarised in **Table 1**. The proposed work was carried out as per the Indian Council of Medical Research (ICMR) guidelines for Approval of Research Activity involving Human Subjects and was approved by the Institutional Ethics Committee (IEC/CBPBU/200/2020/001, MJNMC/IEC/77/2024 and MJNMC/IEC-78/2024). Informed and written consent were obtained from each subject participating in the study as per the ICMR guidelines.

**Table 1:** Clinical data of human subjects.

Patient characteristics	Normal pregnancy (n=6)	Preeclampsia (n=6)	Gestational diabetes mellitus (n=4)	Recurrent pregnancy loss (n=4)
Age, Year	25.2 ± 4.27	25 ± 4.63	26 ± 4	26 ± 4
Gestation age at delivery, week	≥36	≥34 (n=1) ≥36 (n=5)	≥36	≥7-14
Number of pregnancies	1-2	1-2	1-2	2-3
Maximum proteinuria, mg/mmol	–	≥+3	Trace protein	--
Maximum systolic pressure, mm Hg	≤120-140	≥150-178	≥138-160	≥100-140
Maximum diastolic pressure, mm Hg	≤80	≥110	≥84-100	≥70-80
New-born weight, g	2840-3200	1680-3100	2240-4100	--
Blood sugar (mg/dl)	98.83 ± 7.83	88.33 ± 8.66	198 ± 18.56	105.5 ± 12.23
Previous abortion history	Nil	Nil	Nil	2-3

Human placental (including chorionic villi and decidua) and umbilical cord tissues were collected within an hour following the delivery. They were dissected (1cm) into small pieces (five different sample sites across each placenta), kept in a vial containing phosphate buffered saline (PBS), and immediately transferred to the laboratory (CBPBU) in a cold storage container. For group1/PE, group 2/GDM and group 4/N, third trimester (term end) placentae were collected; for group 3/RPL, first trimester (early term) placentae was collected. Tissues were washed thoroughly 2 to 3 times in fresh PBS to remove blood. For gene expression profiling, tissues were kept overnight in RNA Liv (HiMedia) solution; next day, RNA Liv was discarded and tissues were kept at -80°C. For protein expression profiling and biochemical analysis, tissues were snap-frozen and stored at -80°C, and for histopathology and immunohistochemistry, tissue samples were formalin-fixed.

### RNA isolation and reverse transcriptase quantitative polymerase chain reaction (qPCR)

Following the manufacturer’s instructions, 40 mg of placental and umbilical cord tissues were homogenised in RNA Xpress (HiMedia) and centrifuged at 12000×g for 10 min and the RNA containing supernatant was collected. Initially, total RNA was isolated using chloroform (0.2ml v/v), and then precipitated using isopropanol (0.5ml v/v) by centrifuging at 15000×g at 4°C. Next, the pellet containing RNA samples were treated with RNase free DNase I (GeNei™, 065560001A), extracted with chloroform, and precipitated with isopropanol in order to prevent the contamination with genomic DNA. After being cleaned with 75% ethanol and resuspend the RNA pellet in nuclease-free water, RNA was quantified using NanoDrop^®^ spectrometer (Thermo Scientific, NanoDrop one) at 260 nm. First strand cDNA was synthesized using the iScript Reverse Transcription Supermix (Bio-Rad, 1708841) with 1ng of total RNA. The resulting cDNA was subjected to qPCR with SsoAdvanced Universal SYBR^®^ Green Supermix (Bio-Rad, 1725270) using the Biorad CFX Maestro (CFX Connect™ Real-Time System). 40 cycles of denaturation (30 sec at 95°C) and annealing (30 sec at 54.6°C to 57.2°C, annealing temperature of the primers) were carried out. Housekeeping gene (β-actin) expression was used to normalise the data. After normalizing with β-actin abundance in the same sample, the ΔΔCT comparative technique was used to semi-quantitate the relative target mRNA (85). **Supplementary Table 1** provides specific primer sequence information, product size, annealing temperature, and primer pair sequences.

### Protein isolation from placental tissue and umbilical cord and immunoblot analysis

Protease inhibitor cocktail (ML051; HiMedia) and RIPA lysis buffer were used to homogenise placental and umbilical cord tissues (~50 mg each). Following homogenisation for 10 min, the samples were centrifuged for 20 min (14,800 rpm) at 4°C. Using Folin-Lowry’s method, the total protein content in the supernatant was measured. 40µg of protein was loaded on to each well and resolved under reducing conditions using 10% v/v SDS-PAGE for properdin and FH, and 8% v/v SDS-PAGE for C3 and C5. The proteins that have been resolved were wet transferred to a nitrocellulose membrane (Biorad). The membrane was then blocked for 45 min at room temperature using 2% BSA (bovine serum albumin) (Sigma Aldrich) in TBST [Tris-buffered saline containing 0.1% (v/v) Tween-20]. The membrane was probed overnight at 4° C with their respective primary antibodies. The following day, the blots were washed in TBST and probed for 2 h with HRP-conjugated secondary antibodies at room temperature. Finally, the protein bands were revealed using the Immobilon substrate (Millipore) via ChemiDoc machine (Biorad). Quantitative densitometric analysis was carried out via ImageJ software. Antibody details, blocking reagents and dilutions are summarised in **Supplementary Table 2**.

### Immunohistochemistry

For Haematoxylin and Eosin (H&E) and Masson’s trichrome (MT) staining, 1cm thick sections of placental (sampled from the marginal zone bordering the yolk sac) and umbilical cord tissues were processed through ethanol gradient (70%→80%→90%→99% ethanol), cleared in xylene (Merck) and infiltrated with paraffin at 60°C to prepare the paraffin block. A manual rotary microtome (Leica) was used to slice 4 μm thin tissue sections of the paraffin-embedded samples. After stretching the tissue samples on a glass slide, it was deparaffinized in xylene and rehydrated in graded ethanol followed by haematoxylin (Rankem) staining and then dehydrated. The tissue samples were then stained with eosin (SRL), washed in ethanol, dealcoholized, cleared in xylene and mounted using DPX (di-butyl-phthalate polystyrene xylene). MT staining was carried out with Anneline blue (SRL) to study collagen deposition. H&E and MT-stained tissues were photographed via bright field microscope (Axiolab 5, ZEISS).

For IHC, 4 µm sliced tissue sections were placed on poly-L-lysine coated glass slides. Tissue sections were deparaffinizing in xylene and then rehydrated progressively, using 90%, 70%, 50%, 30% (v/v) ethanol and distilled water. After 30 min of endogenous peroxidase blocking with a 3% v/v hydrogen peroxide solution, the slides were replaced in sodium citrate buffer, pH 6.0 (Thermo Fisher Scientific) and microwaved for antigen retrieval. The tissue sections were blocked in 5% w/v BSA for 1 h at room temperature and incubated overnight with mouse anti-human properdin monoclonal antibody (0.9mg/ml in PBS; 1:200 dilution in 1% BSA in PBS) and sheep anti-human FH polyclonal antibody (1:500 dilution in 1% BSA in PBS) at 4°C in a humidified chamber separately. Next day, the slides were washed with PBS, incubated with HRP-conjugated goat anti-mouse IgG (H+L) (1:1000 dilution in 1% BSA in PBS) for properdin and HRP-conjugated rabbit anti-sheep IgG (H+L) (1:1000 dilution in 1%BSA in PBS) for FH for 2 h in a moist chamber at room temperature. The sections were counter-stained with haematoxylin (Rankem) after being treated with DAB (3,3-Diaminobenzidine) (SRL) substrate for three min in the dark. Slides were mounted using Dibutylphthalate Polystyrene Xylene (DPX) and observed under phase contrast microscope (Zeiss). Images were captured at 10X magnification. The amount of immunostaining was measured using a digital image analysis tool, the open-source IHC profiler plug-in for ImageJ (86, 87).

### Statistical analysis

For statistical analysis, non-parametric Mann-Whitney test was performed in between control (N) versus PE, control (N) versus GDM and control (N) versus RPL for RT-qPCR and WB. For IHC, non-parametric Mann-Whitney test was performed in between control (N) versus PE, control versus (N) GDM and PE versus GDM. The data were displayed as the average ± standard deviation of the mean (SD). For each analysis, the results were shown as statistically significant, when *p <0.05, **p <0.01, *** p<0.001 and ns=not significant.

## Results

### High mRNA expression of properdin in PE and GDM placentae, whereas low expression of FH in PE and high in GDM placentae compared to normal healthy placentae

**Figure 1:**
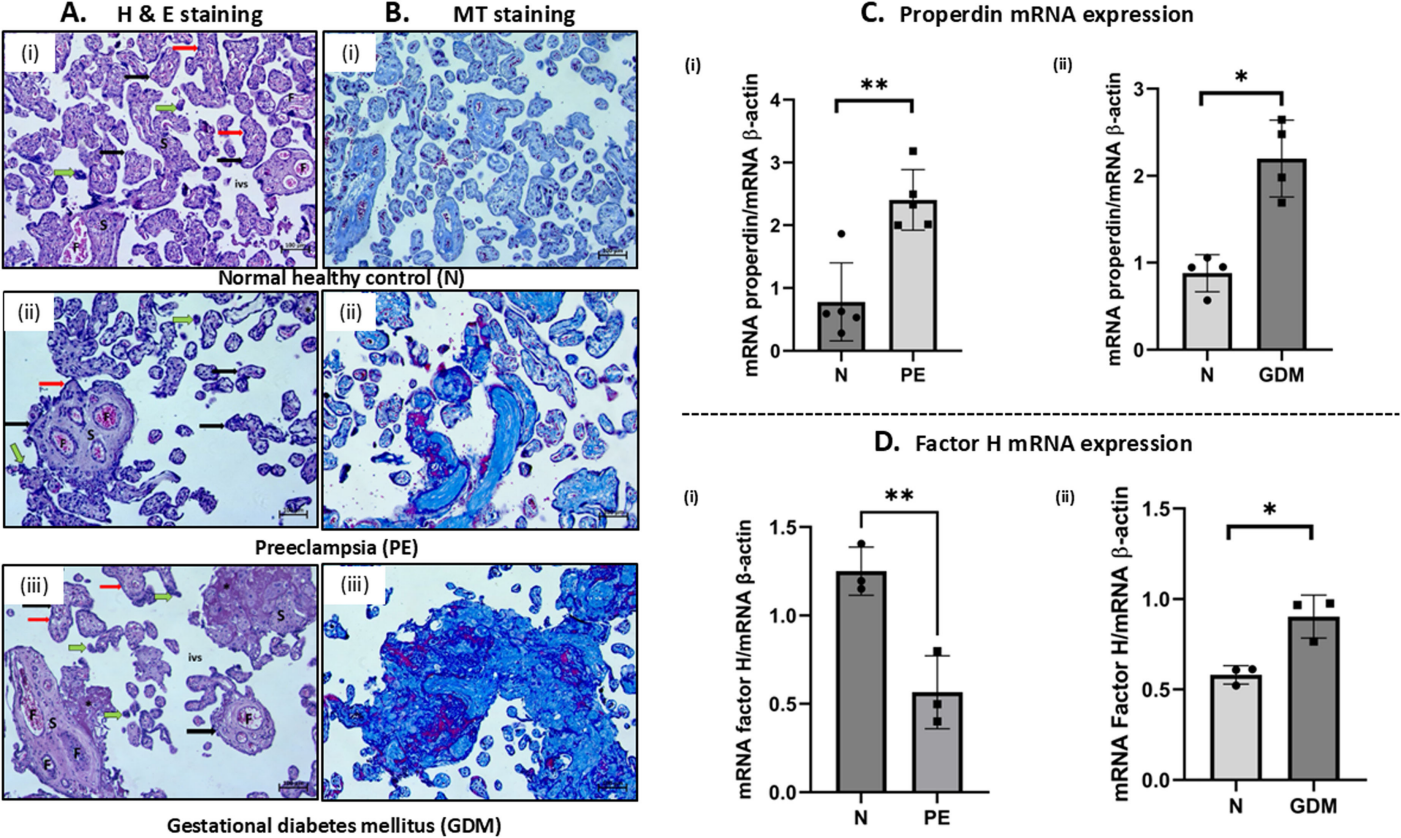
**(A)** Haematoxylin and eosin (H & E) staining of the placental tissue. (i) normal healthy placenta (N) showed foetal villi (black thick arrow) surrounded by syncytiotrophoblast layer (red arrow) separated by intervillous spaces (ivs) and villous stroma (S) containing fetal blood capillaries **(F)**, (ii) Thickened fetal blood capillaries **(F)**, syncytial knots (green arrows) and fibrinoid deposition (*) were observed in PE placenta. (iii) GDM placenta also showed excessive fibrinoid deposition (*) and thickened fetal blood capillaries **(F)**. Scale bars 100μm. **(B)** Masson’s trichrome (MT) staining microphotographs of the same sample group (i) Light blue colour indicates delicate collagen deposition in N (ii, iii) where dark blue colour indicates dense collagen deposition around the fetal blood capillaries and inside the villous stroma in the PE and GDM placental tissues. Scale bars 100μm. **(C)** mRNA level of properdin was measured by RT-qPCR in N, PE and GDM. (i) PE (n=5) placentae showed significantly high properdin mRNA expression as compared to the N (n=5). (ii) GDM (n=4) placentae also showed significantly high properdin mRNA expression as compared to N (control) (n=4). **(D)** FH mRNA levels were analysed in PE, GDM and N placental tissue; (i) PE (n=3) showed significantly low FH mRNA levels as compared to N (n=3). (ii) GDM (n=3) showed significantly high FH mRNA level as compared to N (n=3). RT- qPCR of properdin and FH gene in PE, GDM and N was normalized with β-actin (housekeeping gene). (*p<0.05, **p<0.01, ns, non-significant).

Initially, to confirm the histopathological characteristics in PE and GDM, H&E and MT staining of placental tissue were carried out. The H&E results showed term placenta with thickened foetal blood capillaries, excessive fibrinoid deposition and a considerable number of syncytial knots in the placental tissue of PE and GDM as compared to N placentae (**Figure 1A**). Earlier studies have demonstrated a substantial collagen deposition in PE placentae which is associated with fibrosis, one of the well-known pathological attributes of PE (88–90). This collagen deposition in the sample groups was also examined via MT staining. A characteristic of PE is maladapted spiral artery remodelling, which is shown by the loss of smooth muscle (red stained) and its replacement by collagen (blue stain). Dense collagen deposition (blue stained) around the foetal blood capillaries and inside the villous stroma of PE and GDM placentae compared to N placentae were observed (**Figure 1B**).

In the RT-qPCR analysis, a significantly higher level of properdin transcript was observed in PE and GDM placentae as compared to N (**Figure 1C**i, ii). Conversely, a significantly low expression level of FH transcript in the PE was noted (**Figure 1D**i), whereas, the FH mRNA gene expression level in the GDM placentae was significantly higher compared to the N placenta (**Figure 1D**ii).

### High mRNA expression of properdin in the umbilical cord of PE and GDM, whereas low FH mRNA expression in PE and high in GDM compared to normal healthy placentae

**Figure 2:**
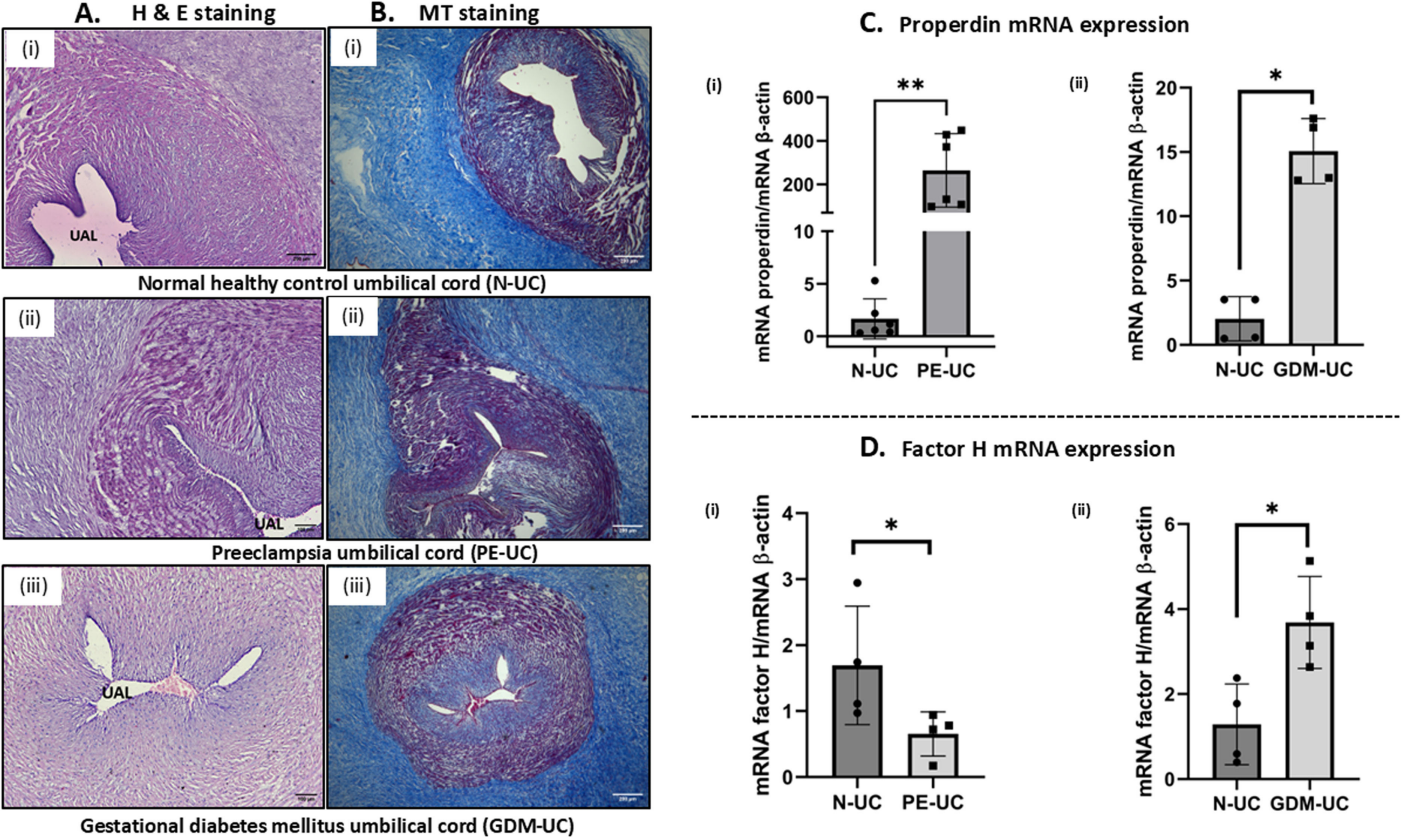
**(A)** H & E staining of the umbilical cord (i) N-UC, ii) PE-UC, iii) GDM-UC. Normal umbilical arterial lumen (UAL) where observed in N-UC, whereas PE-UC and GDM-UC showed narrow umbilical arterial lumen (UAL). Scale bars 100μm **(B)** MT staining of the umbilical cord of three sample groups (i) N-UC, (ii) PE-UC and (iii) GDM-UC with no marked differences in the collagen deposition. Scale bars 200μm **(C)** mRNA expression of properdin through RT-qPCR (i) PE (n=6) (ii) GDM (n=4). Properdin transcript indicates significantly high expression in PE and GDM umbilical cord as compared to control (N-UC). **(D)** Expression of FH transcript in the umbilical cord of (i) PE (n=4), significantly low expression of FH transcript in PE-UC, (ii) GDM (n=4), significantly higher expression of FH mRNA level in GDM-UC compared to control (N-UC). β-actin was used as a housekeeping gene. (*p<0.05, **p<0.01, ns, non-significant).

In addition to PE and GDM placentae, we examined the histology of umbilical cord, which revealed narrow umbilical arterial lumen (UAL) compared to the control (N-UC) (**Figure 2A**). A large gap in the Warton’s jelly was also observed in PE-UC, which indicates oedema-like pathological condition. MT staining of PE/GDM-UC did not reveal any difference in the collagen intensity compared to N-UC (**Figure 2B**).

Properdin and FH transcript levels in the PE-UC and GDM-UC were examined via RT-qPCR. The results showed significantly high properdin transcript expression in the PE and GDM in contrast to the normal healthy UC (N-UC) (**Figure 2C**i, ii). As seen in the placentae of PE and GDM, FH mRNA expression significantly decreased in PE-UC (**Figure 2D**i); GDM-UC showed significantly higher FH transcript expression compared to N-UC (**Figure 2D**ii).

### Higher properdin protein expression in placentae of PE and GDM, whereas lower protein expression of FH in PE and higher FH in GDM in the placentae

**Figure 3:**
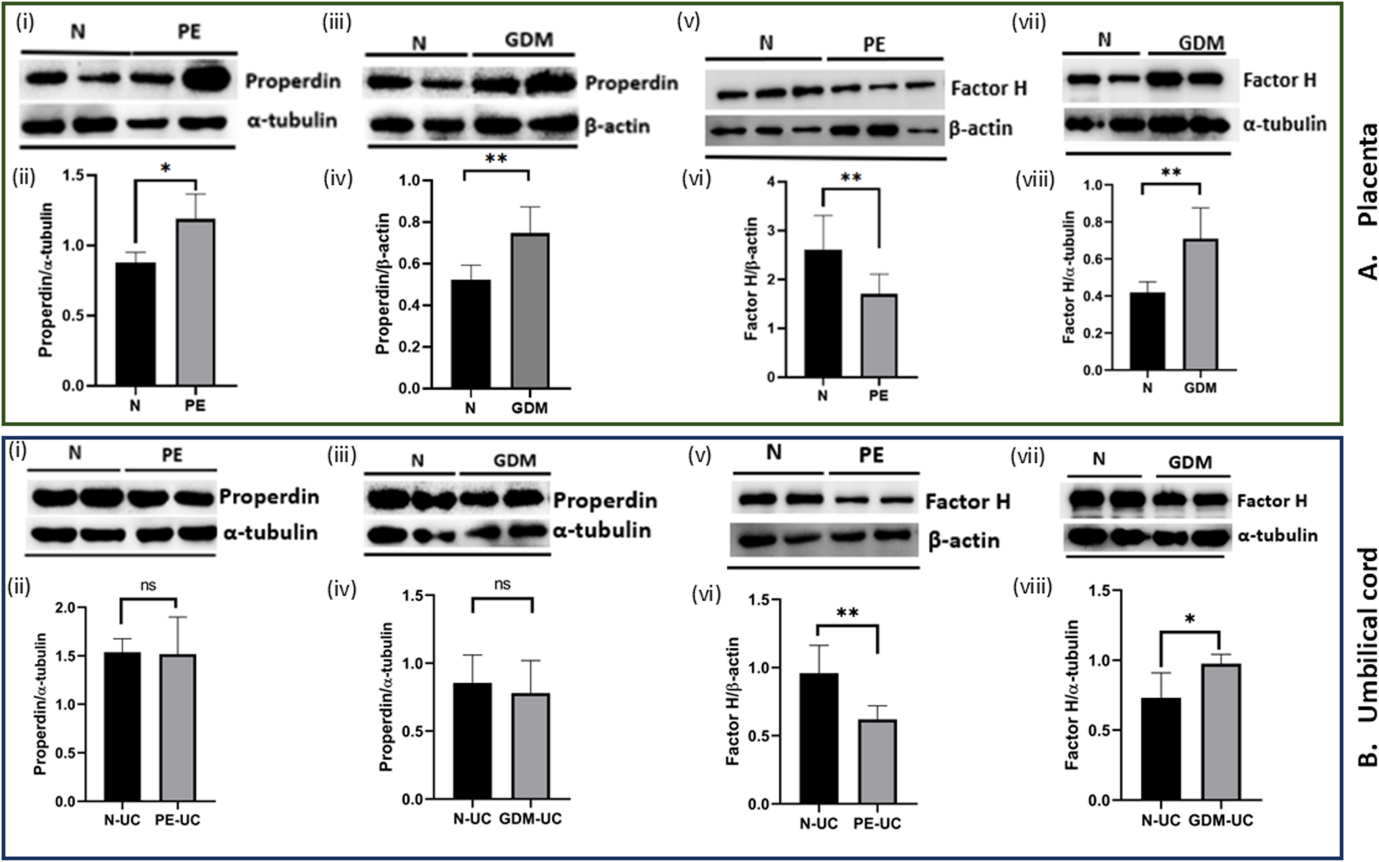
Protein expression of properdin and FH in the placental tissue and umbilical cord of PE and GDM measured through western blot analysis. The densitometry analysis represents biological repeats run on different blots with sample size being n=6 for each N and PE and n=4 for GDM. **(A)** (i, ii, iii, iv) Properdin protein expression in the placental tissue. Both PE and GDM placentae showed significantly high properdin expression as compared to normal healthy placenta (N). (v, vi, vii, viii) FH protein expression in the placental tissue of PE and GDM. Significantly, lower level of FH in PE and higher level of FH in GDM compared to N. **(B)** Properdin and FH protein expression in the umbilical cord of PE (n=4) and GDM (n=4) as compared to N (n=4). (i, ii, iii, iv) No significant differences were observed in properdin protein expression of PE-UC and GDM-UC as compared to N-UC. (v, vi) In PE-UC significantly decreased level of FH were observed as compared to N-UC, (vii, viii) the FH protein level was significantly higher in GDM-UC compared to N-UC. Densitometric analysis of proteins was normalized with α-tubulin and β-actin. Signal intensity was detected using open-source ImageJ software. (*p<0.05, **p<0.01, ns, non-significant).

The expression of properdin and FH at the protein level was assessed by WB. The results were consistent with RT-qPCR data, revealing significantly higher properdin expression in PE (**Figures 3A**i, **3A**ii) and GDM (**Figures 3A**iii, iv) placentae as compared to N. However, FH protein level was significantly low in PE (**Figures 3A**v, vi), while higher in GDM (**Figures 3A**vii, viii). Therefore, the results corroborate the gene expression results.

We also investigated the properdin and FH protein expression in the UC of term sample groups (PE, GDM and N). Protein expression of properdin did not vary among the PE-UC (**Figures 3B**i, ii) and GDM-UC (**Figures 3B**iii, iv) compared to N-UC. However, FH protein expression followed the similar trend, being significantly lower in PE (**Figures 3B**v, vi) and higher in GDM (**Figures 3B**vii, viii) compared to N-UC.

### Localization via IHC of properdin and FH in the PE and GDM placental tissues

**Figure 4:**
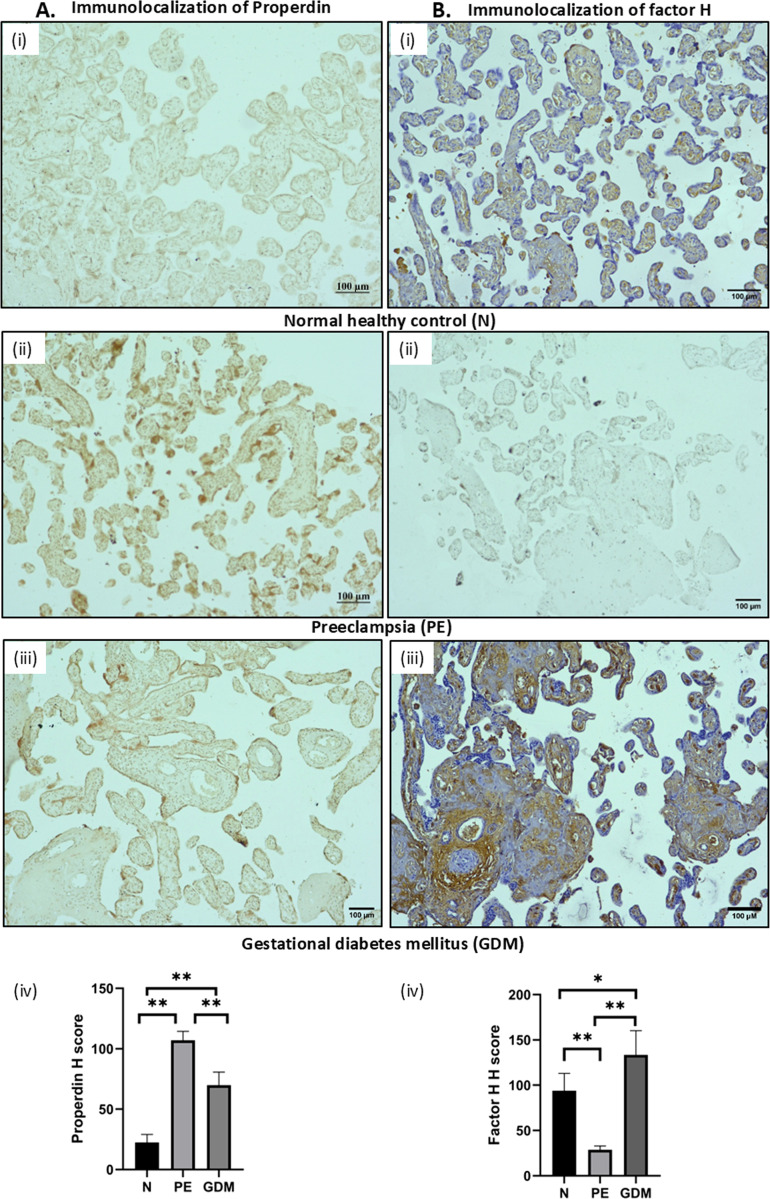
**(A)** IHC images of properdin immunolocalization in N, PE and GDM. Sections were stained with anti-properdin polyclonal antibody. (i) N, (ii) PE and (iii) GDM. The deep brown colour in PE and GDM placenta indicates significantly high properdin expression compared to N. Scale bars 100μm. **(B)** IHC images of FH immunolocalization in (i) N, (ii) PE and (iii) GDM. Sections were stained with anti-FH polyclonal antibody. Scale bars 100μm. Significantly high FH expression in GDM and N placenta compared to PE. Anti-FH staining was very low in PE placenta. Staining was detected with 3,3’-diaminobenzidine (DAB) and counter stained with haematoxylin. For properdin n=3 and for FH n=4 for each group (N, PE and GDM). **(A)**iv) and **(B)**-iv) Bar graph indicating the average H score (IHC intensity score) of properdin and FH of N, PE and GDM. (*p<0.05, **p<0.01, ns, non-significant).

To assess the expression along with the localization of properdin and FH in the placental tissues of PE and GDM compared to the N, immunohistochemistry (IHC) was performed. PE, GDM and N placentae were probed with anti-human properdin and anti-human FH antibodies separately; the positively stained sites showed deep brown colour. Properdin expression was observed in all placental types, but at different intensities. In comparison with the N placentae (**Figure 4A**i), the properdin expression was higher in both the PE (**Figure 4A**ii) and the GDM (**Figure 4A**iii) placentae. Higher expression was observed throughout the syncytiotrophoblast layer and in the syncytial knots (**Figure 4A**). FH expression was very low, almost undetectable in PE placentae (**Figure 4B**ii); in N (**Figure 4B**i) and GDM placentae (**Figure 4B**iii), FH was found to be localized in the syncytiotrophoblast region, in the foetal villous endothelium as well as in the villous stroma region (**Figure 4B**). The IHC results revealed significantly higher properdin (**Figure 4A**iv) and lower FH (**Figure 4B**iv) expression in PE in comparison to N. Unlike PE, in GDM placentae, both properdin (**Figure 4A**iv) and FH levels (**Figure 4B**iv) were higher compared to N. Our IHC results corroborated the RT-qPCR and WB results, which revealed higher properdin expression in both PE and GDM placentae, and significantly lower FH expression in PE and high in GDM compared to N, suggesting association of properdin and FH levels with PE and GDM-associated pathologies.

### Higher expression of key complement components, C3 and C5, in PE placentae, whereas low C3 level in the umbilical cord of PE and GDM

**Figure 5:**
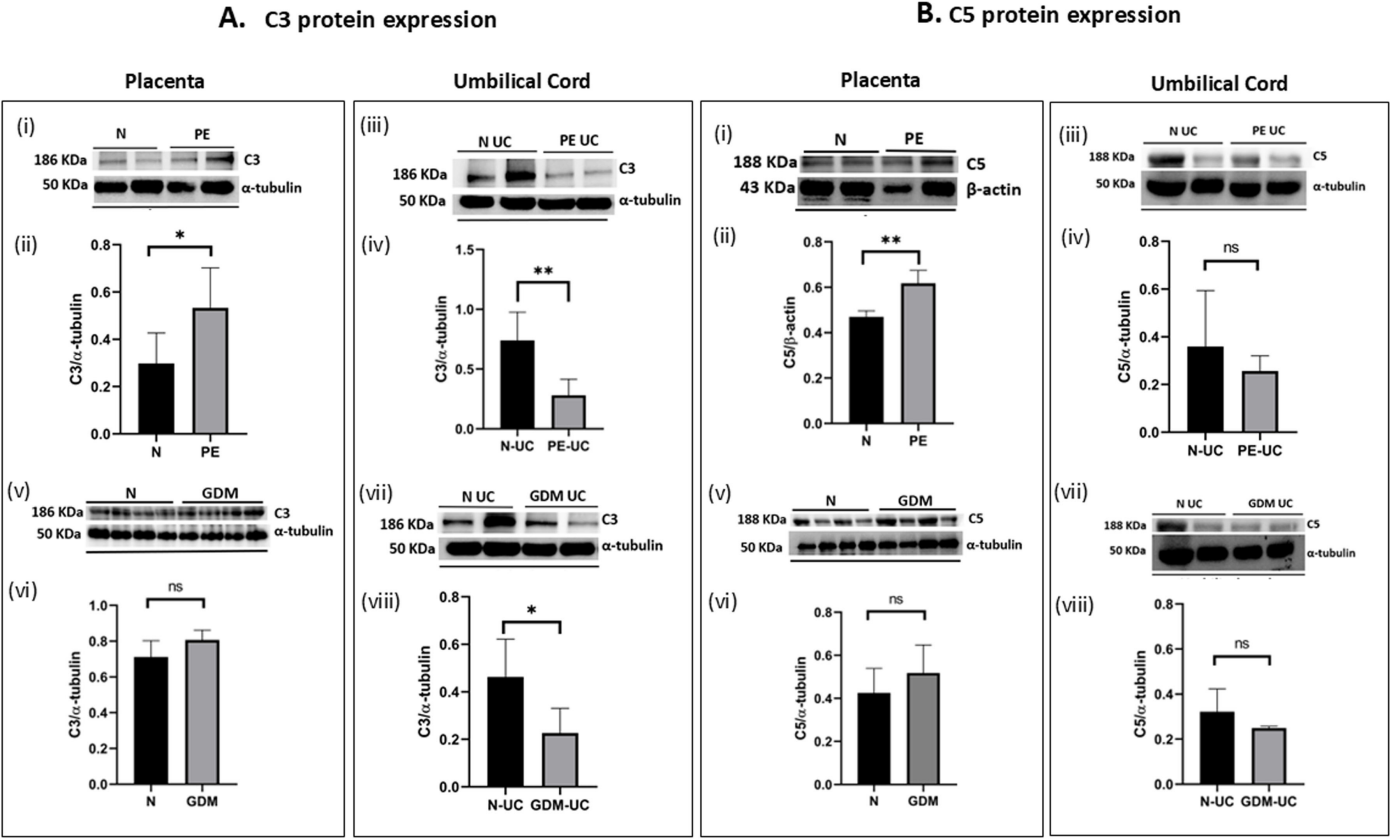
Differential expression of C3 and C5 protein in the placental tissue and umbilical cord of PE and GDM measured through western blot analysis. The densitometry analysis represents biological repeats run on different blots **(A)** C3 protein expression in (i, ii, v, vi)placental tissue of PE (n=6) and GDM (n=4) compared to N (n=6). Significantly increased level of C3 were observed in PE. In GDM there was no significant difference in C3 expression with N. C3 protein expression in (iii, iv, vii, viii)umbilical cord (n=4) of PE, and GDM (n=4) compared to N. Both PE and GDM umbilical cord showed significantly low C3 expression as compared to normal umbilical cord (N-UC). **(B)** C5 protein expression in (i, ii, v, vi)placental tissue of PE (n=6) and GDM (n=4) compared to N (n=6). In PE placentae, increased level of C5 was observed as compared to normal healthy placentae, (iii, iv, vii, viii)umbilical cord of PE (n=4) and GDM (n=4) as compared to N (n=4). Result showed no significant difference in C5 protein expression in PE-UC and GDM-UC compared to N-UC. Densitometric analysis of proteins were normalized with α-tubulin and β-actin. Signal intensity was detected using open-source ImageJ software (*p<0.05, **p<0.01, ns, non-significant).

In the case of PE, both the C3 (**Figures 5A**i, ii) and C5 (**Figures 5B**i, ii) protein expressions in placentae were significantly higher compared to the N placentae, suggesting complement overactivation in the PE placenta. GDM placentae did not show any significant differences in C3 and C5 expression compared to N (**Figures 5A**v, **5A**vi, **5B**v, **5B**vi).

In umbilical cord tissue, the complement protein expression maintained a similar pattern in both PE and GDM. C3 protein expression was significantly lower over the control (N-UC) in both PE-UC (**Figures 5A**iii, iv) and GDM-UC (**Figures 5A**vii, viii). Unlike placentae, C3 level was low in the umbilical cord tissue of PE. However, no differences in the expression of C5 were observed in PE-UC (**Figures 5B**iii, iv) as well as GDM-UC (**Figure 5B**vii, viii) when compared to N-UC.

### Higher protein expression of properdin, C3 and C5, and lower FH protein in first trimester RPL placentae

**Figure 6:**
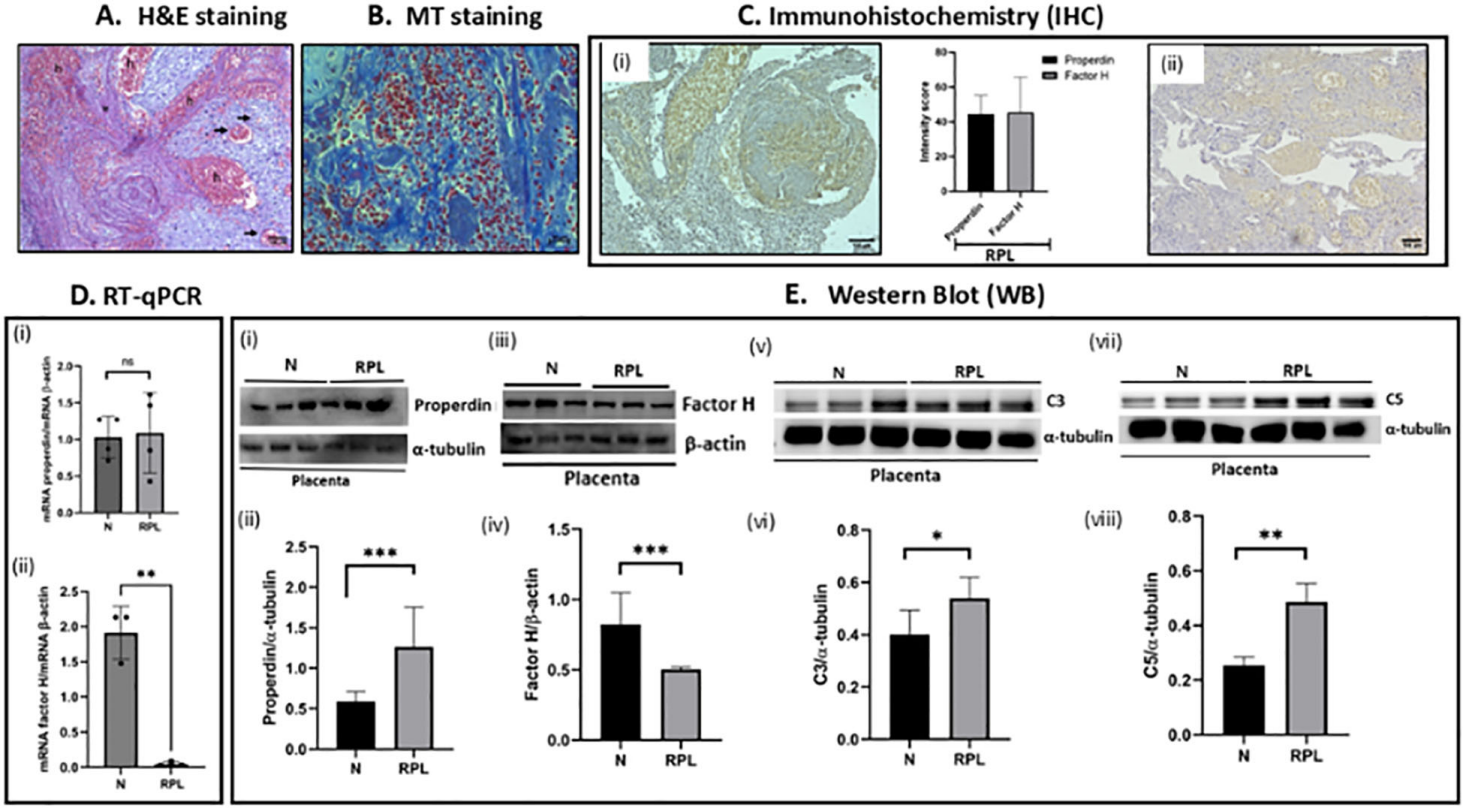
Representative histological photomicrograph of the placental tissue of first trimester recurrent pregnancy loss (RPL). The densitometry analysis represents biological repeats run on two different blots **(A)** H&E-stained RPL placental tissue showed dilated blood vessels in the decidua (black arrow), fibrin deposition (*) and abundant fresh intervillous haemorrhage **(h)**. Scale bars 100μm. **(B)** MT staining of the same RPL sample stained dark blue, indicating dense collagen deposition. Scale bars 20μm. **(C)** Immunolocalization of (i) properdin and (ii) FH in RPL. Presence of both properdin and FH was observed in the RPL placenta with similar intensity. The intensity was quantified by an open-source plugin, IHC profiler compatible with open-source digital image analysis software ImageJ followed by calculation of H score. Scale bars 100μm. **(D)** Expression of properdin and FH mRNA in the placental tissue of RPL (1^st^ trimester) as compared to N (3^rd^ trimester) through RT- qPCR. β-actin used as a housekeeping gene. (i) no significant difference in the mRNA expression of properdin was observed in RPL compared to N (ii) FH expression was significantly very low in RPL compared to N **(E)** Expression of properdin, FH, C3 and C5 protein in RPL (1^st^ trimester) as compared to N (3^rd^ trimester) were measured via western blot. The densitometry analysis represents biological repeats run on different blots. (i, ii) Properdin protein expression showed significantly higher in RPL (n=4) as compared to N (n=4). (iii, iv) FH showed significantly low protein expression in RPL (n=3) as compared to N (n=3). Protein expression of (v, vi) C3 and (vii, viii) C5 were significantly high in RPL (n=4) as compared to N (n=4). Densitometric analysis of proteins were normalized with α-tubulin and β-actin. Signal intensity was detected using open-source ImageJ software. (*p<0.05, **p<0.01, ***p<0.001, ns, non-significant).

The 3^rd^ adverse pregnancy group considered in this study was recurrent pregnancy loss (RPL); here the available placental tissue was at its first trimester decidua. Due to the unavailability of the matched control placenta, RT-qPCR and WB results of RPL were compared with 3^rd^ trimester healthy placental tissues (N) that were used as a healthy control for PE and GDM. The H&E staining of RPL showed dilated blood vessels in the decidua, fibrinoid deposition and intervillous haemorrhage (**Figure 6A**). MT staining revealed dense collagen deposition (blue stain) throughout the placental section (**Figure 6B**). Due to unavailability of the control tissue as mentioned above, IHC analysis comparison couldn’t be done with N tissue. Our IHC staining revealed presence of properdin (**Figure 6C**i) as well as FH (**Figure 6C**ii) inside the chorionic villi and decidual vessels (**Figure 6C**). In our earlier report, we have shown the presence of FH in the syncytiotrophoblasts of 1^st^ trimester healthy placenta (56).

At the protein level, significantly increased properdin expression (**Figures 6E**i, ii) in RPL was found in comparison to N, together with C3 (**Figures 6E**v, vi) and C5 (**Figures 6E**vii, viii) protein expression, indicating more complement activation in RPL. Following the same pattern as PE, significantly low levels of FH mRNA (**Figure 6D**ii) and protein (**Figures 6E**iii, iv) were observed in RPL, indicating dysregulated complement activation in RPL similar to PE.

## Discussion

Complement system is one of the primitive multi-tiered interacting pathways that is a crucial part of the innate immunity in humans. This complex complement proteolytic cascade can be triggered, initially by engaging with soluble pattern recognition molecules, off shooting bioactive fragments that act as anaphylatoxins and generating MAC, controlling the infectious non-self. The bioactive fragments generated through complement pathways are capable of binding complement receptors expressed on a range of cell types to mediate diverse cellular and molecular responses. During pregnancy, in order to safeguard the foetus, complement regulators at the feto-maternal interface maintain an appropriate complement activation, neither less nor more. With time, the importance of this sentinel system has gained importance, reshaping our understanding to complement pathobiology in pregnancy. In this study, we have considered two important complement regulators, properdin and FH, which if modulated, can fuel the inflammatory response in the placenta risking the life of both mother and the foetus. Healthy term-end (third trimester) placenta was considered as control for comparison with the pathological cases. The limitation of the study is lack of a gestational age-matched placenta, while comparing with RPL placenta. Using a first trimester healthy placenta would have been scientifically ideal as epigenetic remodelling in about 7519 of 12000 genes took place from first to third trimester placenta (91). Ethical constrains precluded the availability of such samples. Thus, we compared the properdin and FH levels in RPL with the available third trimester healthy placenta (control). However, to the best of our knowledge, this is the first study which compares two opposing complement regulators in three adverse pregnancy conditions.

Properdin, which is the only known up-regulator of complement alternative pathway, was significantly high in the placenta at both mRNA and protein level in all the three adverse pregnancy cases, PE, GDM and RPL. Properdin was also significantly high in the umbilical cord of both PE and GDM at mRNA level; however, the expression was not significant at the protein level. This discordance between transcript and protein expression in UC could be due to post-transcriptional or post-translational modifications, or due to protein degradation. To consider samples matching, the gestational age GDM cohort were reduced down to four; considering larger cohort studies might lead to significant findings. The PE and GDM placental tissue considered in the study had thickened foetal blood capillaries and higher syncytial knots compared to healthy placenta. The placental tissues of RPL exhibited dilated blood vessels, fibrinoid deposition and intervillous haemorrhage. PE and GDM placental tissues had excessive and irregular collagen deposition. Fibrotic alterations were frequently seen in PE and GDM placentae, especially in the villous stroma or perivillous fibrinoid regions, which implies tissue remodelling brought about by persistent hypoxia or ischaemia. Excessive collagen deposition in fibrotic villi indicates prolonged ischaemic damage and compromised placental perfusion (88–90, 92). Altered extracellular matrix (ECM) remodelling affects nutrient and oxygen exchange between mother and foetus, and impaired placental function causes poor vascularization or abnormal development of placenta in PE and GDM. Immunohistochemistry results also showed significantly higher level of properdin expression in PE and GDM at the syncytiotrophoblast regions and syncytial knots compared to healthy placentae (N). When compared with GDM, the properdin expression in PE placentae was significantly higher, which possibly explains exaggerated inflammatory condition in PE.

The observed overexpression of properdin in the diseased placentae (PE, GDM and RPL) compared to healthy placentae (N) can be correlated with the local inflammatory environment evident in adverse pregnancies. Immune system dysfunction, particularly a Th1/Th2 cytokine imbalance at the foetal-maternal interface, is observed in PE, GDM and in RPL (93–99). In response to placental ischemia and hypoxia in PE, NK and CD4^+^ T cells are activated, generating pro-inflammatory mediators and reactive oxygen species, which promote the cycle of oxidative stress and endothelial damage (94, 100). TNF-α, IL-6 and IL-17 are among the inflammatory cytokines that are increased in PE, along with persistent immunological activation (101). An imbalance of T-helper cell subsets, with a reduction in regulatory T cells (Tregs) and a rise in Th1 and Th17 cells, further characterizes the pro-inflammatory state in PE (95, 96, 102, 103). In GDM placenta, overexpression of inflammatory markers and hormones exacerbates maternal insulin resistance. The inflammation could spread systemically (throughout the body), contributing to insulin resistance, which is a key problem in GDM. Th1/Th2 cytokine imbalance is caused by increased pro-inflammatory cytokines (e.g., TNF-α, IL-6, IL-8) and reduced anti-inflammatory cytokines (like IL-10), often due to abnormal leukocyte infiltration (104). The metabolic disturbances that cause GDM often continue post-partum and may progress to Type 2 Diabetes Mellitus (T2DM). In GDM, CD68^+^ and CD14^+^ macrophages are increased, suggesting their contribution to inflammation. Cytokines released by these cells (IL-6 and TNF) can trigger inflammation within the placenta (97). In the case of RPL, little is known about its underlying immunological mechanisms; however, predominance of Th1 pro-inflammatory immune response is indicated (99).

In PE placenta, enhanced neutrophil infiltration with higher NETosis has been observed (105). NET-derived cell-free DNA, complexed with myeloperoxidase (MPO; a neutrophil granular protein), was was considerably higher in PE serum compared to healthy mothers that gradually increased towards the term end (106). Similarly, neutrophil infiltrates were higher in the chorionic villi of GDM mothers in their median gestational age when compared to healthy mothers at term end. This study also showed that neutrophils isolated from GDM mothers exhibited greater tendency to perform NETosis and high glucose condition can further promote pro-NETosis activity (107). Interestingly a recent study also suggested an increase in the NETosis markers (MPO, H2A and H2B) in RPL mothers that might contribute to its pathology (108). It is important to note that MPO degranulation from activated neutrophils can influence properdin-mediated complement alternative pathway (109). Neutrophils are a key extrahepatic and local source of properdin (110), which is secreted upon stimulation by inflammatory signals. Properdin amplifies the alternative pathway, enhancing immune responses against pathogens. Properdin can offer a platform for de novo assembly of C3 convertase, which will go on to activate the alternative pathway. Properdin binds and stabilises C3 convertase and makes it easier for C3b to engage with factor B, which causes C3bBb to assemble on the cell surface and mediate complement activation (111). Properdin can also initiate the alternative pathway activation on neutrophils and platelets attached to NETs and enhance C5a generation, by stabilizing convertase and acting as a positive feedback loop for the alternative pathway amplification (59). This uncontrolled activation of the alternative pathway by neutrophil-derived properdin can worsen inflammation, contributing to autoimmune diseases (e.g., vasculitis, rheumatoid arthritis) and tissue damage (112). In pregnancy, this excessive activation can disrupt embryo implantation and placental development, cause placental ischemia/hypoxia, impair foetal growth, and create a cytotoxic, pro-inflammatory environment by disturbing angiogenic factor balance (104). In our study, the GDM placentae showed no difference in the C3 and C5 protein levels compared to N placentae. This possibly indicates complement-independent function of properdin in GDM. Properdin can mediate both complement-dependent as well as complement-independent functions, aggravating the inflammatory conditions. Properdin can act as an opsonin promoting phagocytic clearance of apoptotic or necrotic target cells independent of complement activation (113). Properdin can directly bind to NKp46, a mature NK cell ligand; *cfp* (properdin gene) silencing can abolish NKp46 reporter cell activation. Properdin-deficient individuals frequently acquire *Neisseria meningitidis* (Nm) infection. However, properdin treatment during Nm infection were found to be dependent on NKp46 (114). In a study involving a non-obese GDM mouse model, a significant increase in CD11b^+^ NK cells was observed in the peripheral blood. After pregnancy, when these GDM mice were intraperitoneally injected with streptozotocin (STZ), intrauterine growth restriction occurred with significant increase in the number of CD27^-^CD11b^+^ NK cells in the decidua with enhanced cytotoxic activity (115). Thus, properdin in PE and RPL could be involved in both complement-dependent and -independent pathways, but in GDM, it could be following complement independent pathway as both C3 and C5 levels were low in the placenta.

The other regulator considered in this study is FH, which mainly controls the complement alternative pathway by preventing excessive immune activation. FH inhibits the formation and promotes the breakdown of C3 and C5 convertases (116). FH can prevent complement-mediated tissue damage (73, 74, 117). Recent research shows that by competing with C1q for binding to targets such as cardiolipin, lipid A, *E. coli* (118), and beta-amyloid, FH also negatively controls the classical pathway (119), thereby reducing inflammation. It is primarily produced in the liver (hepatocytes and Kupffer cells), but also in kidney, spleen, heart, lungs, brain, eyes, pancreas, placenta and adipose tissue (120). In the present study, unlike properdin, which was significantly high in the placenta of all the three diseases (PE, GDM and RPL), FH was high only in case of GDM and significantly low in PE and RPL. In PE placenta, where FH was significantly very low, the C3 and C5 protein levels were significantly high. Other studies have also reported higher level of C3, C3b, C3d, C4d C5a, factor B, MBL, C9 and sC5b-9 in PE (32, 33, 35–39, 41, 42, 44, 121). We have recently demonstrated that FH serum level significantly declines in PE mothers with increase in gestational age (3^rd^ trimester < 1^st^ trimester) (56). This near absence of FH in PE placenta could be one of the likely reasons for dysregulated complement activation. Reduced FH expression in PE can also be due to its mutation. Severe PE is predisposed to develop in five rare variants of the FH gene: L3V, R127H, R166Q, C1077S, and N1176K (122). Additionally, anti-FH autoantibodies (123), FH intake, or the fact that a significant illness load overburdens FH can all contribute to FH reduction (54). In the case of 1^st^ trimester placentae of RPL, FH mRNA as well as protein levels were significantly lower when compared to 3^rd^ trimester healthy placentae (N). FH polymorphism (*CFH* rs1065489 G>T/*CFH* rs1061170 T>C) has been associated with reduced risk of RPL (124). In our study, RPL placentae showed significantly higher C3 and C5 protein levels when compared with term end healthy placenta. Previous studies have pointed towards the role of complement in spontaneous abortion, where high C5a level was observed in maternal circulation while the complement regulators, CD46 and CD55, were found three-fold decreased compared to control (125). Thus, low levels of complement inhibitors in RPL could be one possible reason for higher complement activation. Upregulation of properdin and downregulation of FH in PE and RPL, as observed in this study, could be the causal drivers of pathology or secondary consequences of the inflammatory milieu characteristic of these pregnancy-related disorders. Whilst altered properdin and FH expression may contribute to local complement dysregulation, it is also likely to be amplified by upstream inflammatory processes inherent to PE and RPL. Together, these findings identify a previously uncharacterized properdin-FH imbalance that may contribute to the distinct pathophysiological profiles in PE and RPL.

As opposed to PE and RPL, GDM placenta as well as GDM-UC showed higher FH compared to N. Our result is consistent with the previous report that demonstrated high FH level in GDM compared to non-GDM serum samples collected in the third trimester of pregnancy (126). High FH level in GDM could be for avoiding unwanted complement activation in the placenta as we have observed low C3 and C5 levels in GDM. Thus, the pathological condition may not be related to complement over-activation in our GDM cohorts. In the umbilical cord, C3 protein expression was significantly lower in GDM compared to healthy ones. We have recently shown that in the case of healthy pregnancy, FH mRNA was highly expressed in human umbilical vein endothelial cells (HUVECs) derived from the UC, compared to decidual endothelial cells, extravillous trophoblasts and decidual stromal cells (56). Higher expression of FH in HUVECs is possibly for maintaining tight regulation on complement activation in the UC, which directly communicates with the foetus. In adipose tissue, high level of FH is linked with obesity, inflammation, insulin resistance, and decreased high density lipoprotein (HDL) cholesterol, suggesting a role of FH in metabolic diseases (127). In addition to its well-known role in defending the body against infections, the complement system is also crucial for metabolism. Human metabolic disorders are regularly associated with elevated levels of certain complement proteins. The metabolic syndrome (MetS) is more likely to develop in patients with greater levels of C3 and C4 (128, 129). High blood glucose, high triglycerides (TG), insulin resistance, and diabetes are all linked to elevated C3, which is especially effective in predicting future risk (130–132). Increased expression of C1 components (C1q, C1r, and C1s) in fat cells (adipocytes) in insulin-resistant individuals suggests that complement is activated in response to metabolic stress (133). FH levels increase with obesity, presumably as a defence mechanism against excessive complement activation. Research on human clinical samples showed that human fat tissue produces FH, particularly in stromal cells and, to a lesser extent, in adipocytes. Insulin resistance and worse metabolic health (such as lower HDL and greater obesity markers) are correlated with higher levels of FH. Higher FH expression, however, may aid in lowering detrimental inflammation in subcutaneous fat (127, 134). In a study involving Chinese mothers who were in their early pregnancy, high level of mannan-binding lectin-associated serine protease (MASP1 and MASP2) were found associated with GDM (48). In second trimester GDM mothers, increase levels of C3, C4 and FH were also observed (126, 135). A recent study in women with GDM, who developed PE during mid-pregnancy, showed high factor B (FB), FH and C3 levels in circulation compared to GDM subjects without PE (136). FB, an alternative pathway activator, can compete with FH for binding with C3b. Thus, when FH level declines in GDM patients, FB can activate the alternative pathway, increasing the risk of developing PE in GDM subjects. At the same time, activation of the lectin pathway in GDM could lead to the production of inflammatory mediators that may influence the local expression of properdin. This possibly explains the concurrent high levels of properdin and FH in GDM compared to healthy subjects.

In addition to the unavoidable limitation of unmatched gestational ages for RPL samples, the relatively small sample size for cohorts as well as a lack of functional complement activity assays are the limitations of our study. However, the present work shows how upregulation of the positive regulator properdin coincides with a reduction in the key negative regulator FH in adverse pregnancies. This study highlights the paradigm of dysregulated expression of two vital but diabolically opposite complement regulators at the feto-maternal interface in pathological conditions, offering an opportunity for future mechanistic and functional studies.

## Conclusions

The primary conclusions of this investigation were that properdin, the the only upregulator of complement alternative pathway, showed very high expression in PE, GDM and RPL and could add to the high inflammatory condition of the placenta and contribute to the pathobiology. Lower level of FH in PE and RPL could contribute to dysregulated complement activation in the placenta. In GDM, the pathogenic pathway may not be directly complement activation mediated due to the high presence of FH in the placenta and in the umbilical cord. In GDM, several other factors contributing to insulin resistance may be in crosstalk with the complement proteins accounting for the pathogenesis. Thus, understanding the mechanistic pathway that leads to the differential distribution of properdin and FH in PE, GDM and RPL at the feto-maternal interface will be of paramount for complement-related diagnosis and therapeutics.

## Data Availability

The raw data supporting the conclusions of this article will be made available by the authors, without undue reservation.

## Supplementary material

Supplementary Table 1 Details of human primers:

Supplementary Table 2 Details of antibodies.
