# Supplementary material for: Differential expression of complement Properdin and Factor H in the placentae and umbilical cords of mothers with Preeclampsia, Gestational Diabetes Mellitus and Recurrent Pregnancy Loss
Source: Front Immunol. 2026 Feb 3;16:1731251. doi: 10.3389/fimmu.2025.1731251 (PMC12909585; doi:10.3389/fimmu.2025.1731251)
Supplement: Supplementary Table 1 — Details of human primers: [file Supplementaryfile1.docx]

**Supplementary Table 1: Details of Human primers:**

| **Target Primer sequences** | | **Annealing temperature (^o^C)** | **Product size (bp)** |
| --- | --- | --- | --- |
| ACTB  (β-actin) | Forward primer 5’-ACAGAGCCTCGCCTTTGC-3’ | 54.6 | 528 |
|  | Reverse primer 5’-ATCACGCCCTGGTGCCT-3’ |  |  |
| CFP  (Properdin) | Forward primer 5’-GAATGGGCAGTGCTCTGGAAAG-3’ | 57.2 | 141 |
|  | Reverse primer 5’-TTGGAGCAGGTGACAGAGCAAG-3’ |  |  |
| CFH  (Factor H) | Forward primer 5’-CCCGGGGAAATACAGCCAAA-3’ | 55.1 | 419 |
|  | Reverse primer 5’-TCTGGGAGTAGGAGACCAGC-3’ |  |  |

**Supplementary Table 2: Details of Antibodies**

| **Antibodies** | **Company** | **Dilutions for WB** |
| --- | --- | --- |
| Properdin mouse anti-human monoclonal antibody | HYB 3904, 0.9mg/ml in PBS | 1:2000 (v/v) in 1% BSA in TBST |
| Sheep FH anti-human polyclonal antibody | AbD, Serotec Hercules, CA | 1:10000 (v/v) in 3% BSA in TBST |
| C3 rabbit polyclonal antibody | ABclonal A16781 | 1:2000 (v/v) in TBST |
| C5/C5a Rabbit polyclonal antibody | ABclonal A8104 | 1:700 (v/v) in TBST |
| Rabbit polyclonal antibody against human β-actin | Abcam, ab-16039, | 1:2000 (v/v) in TBS |
| α-tubulin Rabbit monoclonal antibody | ABclonal A6830 | 1:2000 (v/v) in TBST |
| HRP-conjugated Goat anti-mouse IgG (H+L), | ABclonal AS003 | 1:4000 (v/v) in 1% BSA in TBST for properdin |
| HRP-conjugated Rabbit anti-sheep IgG (H+L) | ABclonal AS023, | 1:18000 (v/v) in TBST for factor H |
| HRP-conjugated Goat anti-rabbit IgG (H+L) | ABclonal AS014 | 1:2000-1:5000 (v/v) in TBST for C3, C5, α-tubulin and β-actin. |
